# Ginsenoside Rh2 Regulates PI3K/AKT Signaling, Metabolic Pathways, and the Gut Microbiota for Coronary Heart Disease Therapy

**DOI:** 10.3390/ijms27115133

**Published:** 2026-06-05

**Authors:** Zhuowen Chen, Hanye Wang, Ye Yang, Xiuming Cui, Chengxiao Wang, Yuan Liu

**Affiliations:** 1 Faculty of Life Science and Technology, Kunming University of Science and Technology, Kunming 650500, China; chen8070kust@163.com (Z.C.); wanghanyekust@163.com (H.W.); yangyekm@163.com (Y.Y.); sanqi37@vip.sina.com (X.C.); 2 Key Laboratory of *Panax notoginseng* Resources Sustainable Development and Utilization of State Administration of Traditional Chinese Medicine, Kunming 650500, China; 3 Kunming Key Laboratory of Sustainable Development and Utilization of Famous-Region Drug, Kunming 650500, China

**Keywords:** coronary heart disease, ginsenoside Rh2, network pharmacology, *Panax notoginseng*, PI3K/AKT signaling pathway

## Abstract

This study investigated the molecular mechanisms underlying the therapeutic effects of ginsenoside Rh2 (G-Rh2) in coronary heart disease (CHD) through a network pharmacology approach, focusing on identifying key targets and pathways, including those involved in lipid metabolism, metabolism regulation and anti-apoptotic signaling. A multi-target network pharmacology analysis was performed to predict the pharmacoloigical targets and pathways of G-Rh2. Key molecular interactions were validated by molecular docking. In vivo experiments using CHD rat models were conducted to verify and quantify the effects of G-Rh2 on lipid profiles, myocardial pathology, and gut microbiota composition. G-Rh2 significantly ameliorated CHD in rats by reducing serum cholesterol and triglycerides levels, alleviating myocardial fibrosis, suppressing cardiomyocyte apoptosis, and mitigating tissue damage. Mechanistically, G-Rh2 activated the PI3K/AKT signaling pathway, regulated atherosclerosis-associated metabolic pathways (e.g., pentose phosphate and carbon metabolism), and modulated gut microbiota composition by reducing the abundance of harmful bacteria and increasing beneficial microbial populations, thereby enhancing lipid metabolism and energy balance. This study demonstrates that G-Rh2 alleviates CHD through the synergistic activation of the PI3K/AKT pathway, modulation of key metabolic pathways, and restructuring of gut microbiota. These findings underscore the potential of G-Rh2 as a multi-target therapeutic agent for CHD, offering mechanistic insights into its cardioprotective properties and supporting the broader application of G-Rh2 in cardiovascular drug development.

## 1. Introduction

Coronary heart disease (CHD) is a heart disease caused by vascular stenosis or blockage due to atherosclerotic lesions in the coronary arteries [1], leading to myocardial ischemia, hypoxia, or necrosis [2]. It remains a significant global public health concern [3], influenced by multiple risk factors, including hypertension, hypercholesterolemia, physical inactivity, tobacco use, and dietary imbalances [4]. The primary treatment modalities for CHD encompass both surgical interventions and pharmacological therapies. Surgical approaches include coronary endarterectomy [5], percutaneous coronary intervention (PCI), and coronary artery bypass grafting (CABG) [6], Pharmacological treatments primarily comprise nitrates [7], angiotensin-converting enzyme inhibitors (ACE) or angiotensin II receptor blockers (ARBs), beta-adrenergic antagonists (such as nadolol, atenolol, and bisoprolol), antiplatelet agents or anticoagulants, and statins (including simvastatin, fluvastatin, and rosuvastatin) [8].

Although PCI and CABG can improve survival rates in patients with acute myocardial infarction to over 90% [6], these procedures carry a significant risk of postoperative complications, including restenosis, the no-reflow phenomenon, and stent thrombosis. Moreover, the adverse effects associated with statins and antiplatelet agents further highlight the limitations of current treatment strategies [9,10]. The concurrent use of pharmacological and surgical interventions may also precipitate additional complications, such as pulmonary dysfunction and impaired coronary perfusion following vascular reconstruction, resulting in high rates of myocardial infarction recurrence and increased mortality. Therefore, developing safe and effective pharmacological treatments for CHD remains critically important.

Herbal medicine offers multifaceted advantages for CHD treatment [11], notably through its multi-target regulatory mechanisms that simultaneously address lipid metabolism, oxidative stress, and inflammation [12]. It also plays a role in gut microbiome-host co-metabolic regulation by enhancing the short-chain fatty acid production and reducing intestinal permeability [13]. Panax notoginseng (PN), a classic medicinal herb of the Araliaceae family, is widely used in traditional Chinese medicine for its distinctive ability to promote blood circulation and resolve blood stasis [14]. Modern pharmacological studies have demonstrated that PN possesses antiplatelet aggregation activity [15], anti-atherosclerotic effects [16], and lipid-lowering capabilities [17]. These pharmacological actions contribute to the inhibition of vascular smooth muscle cell proliferation and migration, the reduction in blood viscosity, and the prevention of thrombosis, establishing PN as an important agent in the prevention and intervention of cardiovascular diseases [18]. The bioactivity of PN is attributed to its multi-target, multi-pathway mechanisms, which have garnered considerable interest for their potential cardiovascular protective effects, making PN a major research focus in herbal medicine. Notably, ginsenosides derived from PN—particularly those in blood-activating soft capsule formulation—have been widely used in treating cardiovascular conditions, such as CHD, angina pectoris, and myocardial ischemia [19]. This study specifically examined the active component ginsenoside Rh2 (G-Rh2). G-Rh2 serves as a key bioactive ingredient in cooked PN [20]. It has been shown to promote the proliferation of human umbilical vein endothelial cells by upregulating the VEGF/VEGFR2 signaling pathway, thereby enhancing VEGF secretion and offering a novel mechanism for improving coronary microcirculation. Huang et al. [21] screened out the effective components of PN for the treatment of coronary heart disease via network pharmacology, among which G-Rh2 was included. These findings suggest that G-Rh2 holds significant therapeutic potential for CHD, warranting further investigation into its therapeutic efficacy.

**Figure 1:**
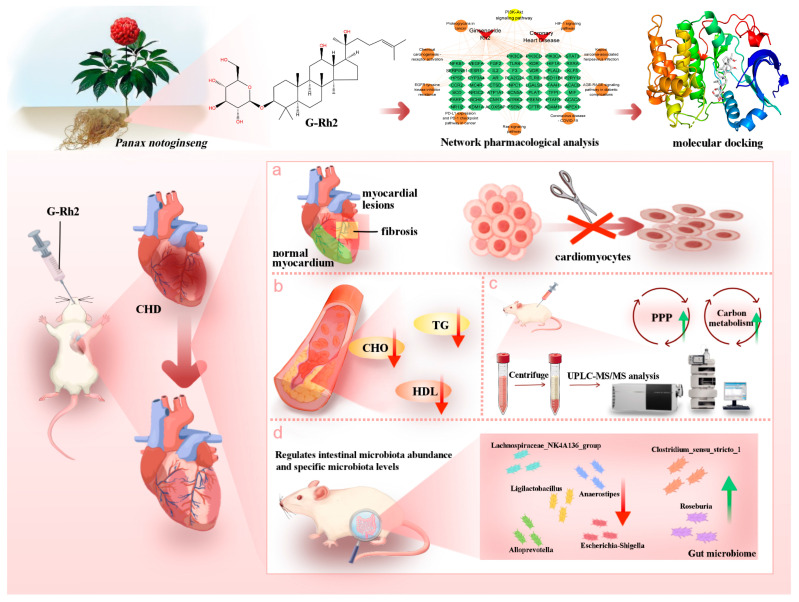
A schematic diagram illustrates the study workflow for elucidating the molecular mechanisms underlying the therapeutic effects of G-Rh2 on coronary heart disease (CHD) through a systems pharmacology approach. Network pharmacology analysis combined with molecular docking validation revealed that G-Rh2 exerts core regulatory effects by targeting the PI3K/AKT signaling pathway. In vivo experiments demonstrated that G-Rh2 markedly reduced serum lipid levels, inhibited cardiomyocyte fibrosis and apoptosis, and attenuated pathological damage in myocardial tissue in a rat model of CHD. Moreover, G-Rh2 activated the PI3K/AKT signaling pathway and modulated atherosclerosis-related metabolic pathways and gut microbiota composition (In the figure, the red "↓" indicates a decrease in content or relative abundance; the green "↑" indicates an increase in relative abundance).

This study employed a network pharmacology approach to elucidate the molecular mechanisms underlying G-Rh2-mediated treatment of CHD (Graphical Abstract). Through multi-target network pharmacology analysis, supported by molecular docking validation, we demonstrated that G-Rh2 attenuates myocardial injury and fibrosis induced by CHD, inhibits cardiomyocyte apoptosis, regulates serum lipid profiles, and exerts core regulatory effects by targeting the PI3K/AKT signaling pathway. Activation of this pathway led to the upregulation of phosphorylated AKT expression, which subsequently initiated downstream cardioprotective cascades. Metabolomics analyses indicated that G-Rh2 exerted its therapeutic effects on CHD primarily by influencing the pentose phosphate pathway, carbon metabolism, and pyrimidine metabolism, while also modulating gut microbiome composition and restoring the intestinal microecological balance (Figure 1). Collectively, these findings identify G-Rh2 as a compound with significant therapeutic potential for CHD, providing a theoretical foundation for the clinical application of PN.

## 2. Results

### 2.1. Network Pharmacology and Molecular Docking

**Figure 2:**
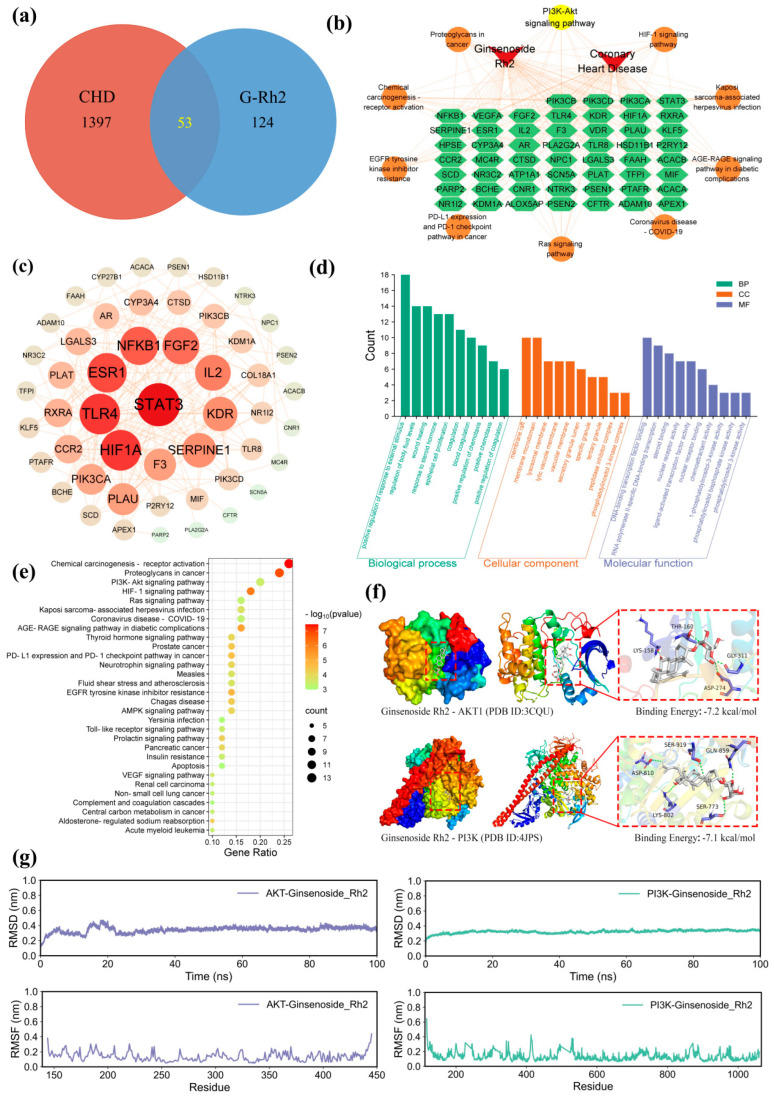
Network pharmacology analysis and molecular docking. (**a**) Venn diagram of target identification; (**b**) “compound–target–disease–pathway” network; (**c**) PPI network; (**d**) GO enrichment analysis; (**e**) KEGG pathway enrichment analysis; (**f**) molecular docking visualization; (**g**) RMSD and RMSF profiles of AKT1 and PI3K complexes with G-Rh2.

A total of 177 potential targets related to G-Rh2 were identified using the SEA, SuperPred, and Swiss Target Prediction databases. By integrating data from the OMIM, HERB, and GeneCards databases, 1450 targets associated with CHD were obtained. Through intersection analysis, 53 potential therapeutic targets for G-Rh2 in CHD treatment were identified (Figure 2a).

Protein–protein interaction (PPI) network and a “compound–target–disease–pathway” network were constructed using the STRING database and Cytoscape 3.9.1 software (Figure 2b,c). Subsequently, 10 core targets for G-Rh2 in CHD treatment were identified from these networks: STAT3, HIF1A, TLR4, ESR1, NFKB1, FGF2, IL2, SERPINE1, KDR, and F3. GO enrichment analysis identified 2608 biological processes (BP), 174 cellular components (CC), and 290 molecular functions (MF) associated with the intersecting targets. The top ten GO terms, ranked by the number of enriched genes, were visualized in a bar chart (Figure 2d), revealing that BP was mainly related to processes such as “positive regulation of response to external stimulus”; CC was primarily associated with membrane rafts and membrane microdomains; and MF was most closely linked to DNA-binding transcription factor binding. KEGG enrichment analysis revealed 121 signaling pathways associated with the intersecting targets. Among these, the top 30 pathways ranked by enriched gene count were selected for KEGG bubble plot visualization (Figure 2e). Although STAT3, HIF1A, and TLR4 exhibited higher rankings in the PPI network based on degree values (Figure 2c), the PI3K-AKT signaling pathway was identified as one of the most significantly enriched pathways relevant to CHD pathophysiology (e.g., apoptosis, inflammation, metabolism) (Figure 2e). Given its well-established central role in cardioprotection and survival signaling, as well as its high enrichment significance, these findings demonstrate that the PI3K-AKT signaling pathway represents a core signaling pathway underlying G-Rh2-mediated CHD treatment.

Molecular docking results (Figure 2f) revealed that the binding energies of AKT1 and PI3K with G-Rh2 were −7.2 and −7.1 kcal·mol^−1^, respectively, both less than −7 kcal·mol^−1^, indicative of strong binding affinity between G-Rh2 and both targets. Visualization analysis revealed that G-Rh2 formed four hydrogen bonds (indicated by green dashed lines) with AKT1 residues Lys-158, Thr-160, Asp-274, and Gly-311, as well as five hydrogen bonds with PIK3 residues Ser-773, Lys-802, Asp-810, Gln-859, and Ser-919. Collectively, these results indicate that G-Rh2 forms more than four hydrogen bond interactions with both AKT1 and PI3K, thereby demonstrating favorable hydrophilic binding characteristics. Furthermore, the binding energies below −7 kcal·mol^−1^ further validate the potential interactions of G-Rh2 with these key targets.

The RMSD curves of the complexes formed by AKT1, PI3K proteins and ginsenoside Rh2 remain steadily around 0.3 nm, which verifies that the constructed complexes possess favorable structural stability from the perspective of root mean square deviation. The fluctuation amplitudes of RMSF curves of AKT1 and PI3K proteins are all less than 1 nm without obvious drastic fluctuations, indicating that the introduction of ginsenoside Rh2 exerts negligible effects on the dynamic stability of amino acid residues in the two proteins, which further confirms the excellent structural stability of the prepared complexes (Figure 2g). Rg analysis revealed steady fluctuations and stable hydrogen bond counts, suggesting favorable hydrogen bonding interactions and high complex stability. SASA analysis indicated high stability for both AKT1 and PI3K (Figure S2). Molecular dynamics simulations confirmed that G-Rh2 consistently binds to the same protein sites with good stability. Free energy analysis (Figure S3) showed that the complexes possessed a single minimum energy cluster, and the calculated binding free energy demonstrated strong binding, with PI3K exhibiting superior affinity. Analysis of binding amino acid residues (Figure S4) revealed that Phe-161, Thr-160, Ile-932, and Met-772 played major roles in binding, with no significant changes observed in the binding sites, thereby confirming the high stability of the complexes.

### 2.2. G-Rh2 Attenuates Myocardial Injury and Fibrosis in CHD

**Figure 3:**
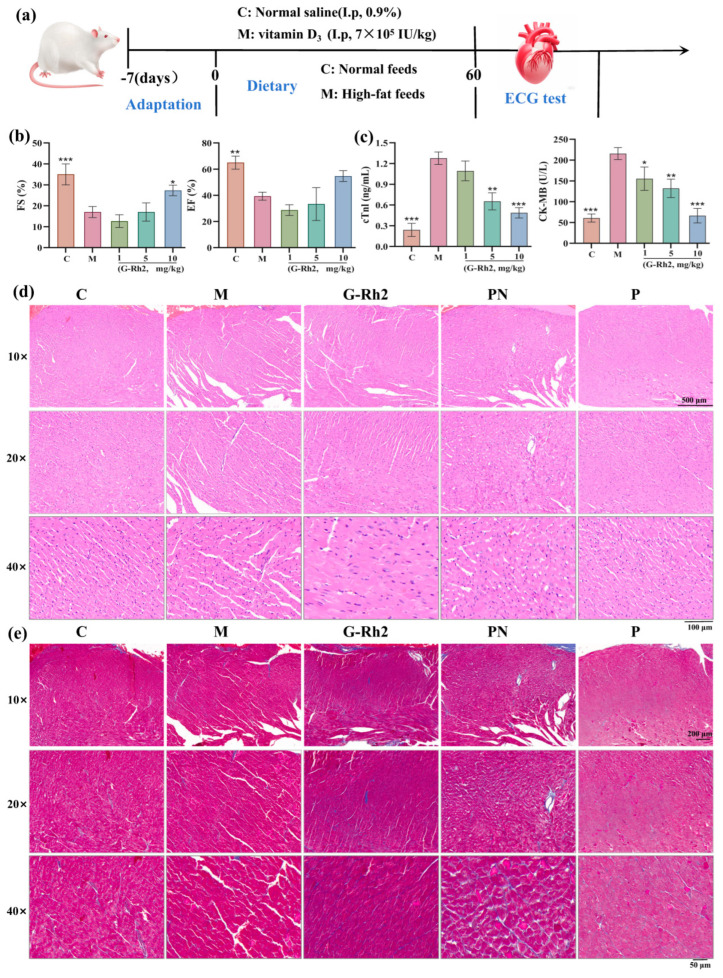
Therapeutic effect of G-Rh2 on CHD in rats. (**a**) Schematic diagram of the CHD model; (**b**) EF (%) and FS (%); (**c**) Serum levels of myocardial injury biomarkers (CK-MB and cTnI); (**d**) Representative images of H&E staining (magnification: 10×, 20× and 40×); (**e**) Representative images of Masson staining (magnification: 10×, 20× and 40×). (C: control group; M: CHD group; G-Rh2: ginsenoside Rh2 treatment group; PN: PN group; P: positive control group). Compared with the M group, * *p* < 0.05, ** *p* < 0.01, *** *p* < 0.001.

In the preliminary dose-finding experiments (Figure 3b), both the left ventricular fractional shortening (FS) and ejection fraction (EF) were significantly decreased (*p* < 0.001) in CHD rats compared with the model (M) group. However, G-Rh2 treatment significantly increased FS (*p* < 0.05) and EF in a dose-dependent manner; specifically, the 10 mg/kg G-Rh2 dose treatment showed superior cardioprotective effects compared to the M group. Additionally, serum levels of the myocardial injury biomarkers CK-MB and cardiac troponin I (cTnI) were quantified by ELISA (Figure 3c). G-Rh2 significantly decreased the CHD-induced elevation of CK-MB and cTnI levels in a dose-dependent manner. Specifically, compared to the M group, 10 mg/kg G-Rh2 produced significant differences (*p* < 0.001) in both CK-MB and cTnI levels. Based on these findings, 10 mg/kg G-Rh2 was selected for subsequent experiments.

H&E staining results (Figure 3d) revealed that myocardial cells in the control (C) group exhibited normal morphology with tightly arranged tissue architecture and no pathological changes. In contrast, the M group exhibited significant pathological alterations characterized by disordered cell arrangement, indistinct cell boundaries, and apoptotic myocardial cells. Compared with the M group, the G-Rh2 group showed significant improvements, including restoration of orderly myocardial fiber arrangement, significant reduction in inflammatory infiltration, marked decrease in myocardial cell apoptosis, and notable alleviation of myocardial tissue damage.

Masson staining results (Figure 3e) demonstrated that myocardial cells in the C group were arranged in regular bundles or spiral patterns, with minimal blue collagen fibers in the interstitium and no apparent fibrotic areas. The myocardial tissue in the M group exhibited distinct fibrotic characteristics, including disordered cell arrangement, cell atrophy, widened intercellular spaces, and a significant accumulation of blue collagen fibers in the interstitium. Collagen fibers formed dense bundles or plate-like structures that replaced native myocardial tissue, consistent with typical CHD pathology. In the G-Rh2 treatment group, notable improvements were observed compared with the M group, characterized by orderly myocardial cell arrangement, reduced blue collagen fibers, decreased myocardial fibrosis, and restored structural integrity.

In conclusion, G-Rh2 exhibits significant cardioprotective effects in attenuating CHD-induced myocardial injury and inhibiting myocardial fibrosis, which may be associated with the regulation of lipid metabolism homeostasis and restoration of myocardial cellular structural integrity.

### 2.3. G-Rh2 Attenuates Cardiomyocyte Apoptosis in CHD

**Figure 4:**
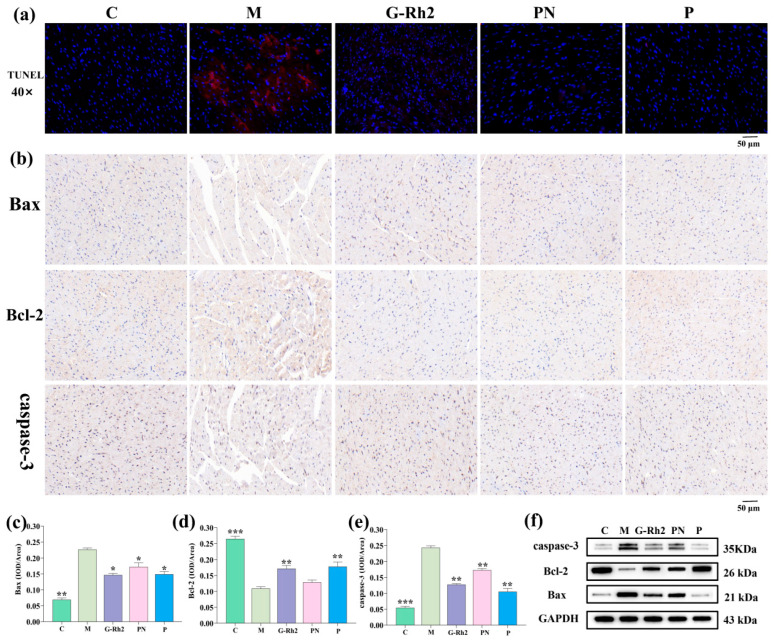
Effects of G-Rh2 on cardiomyocyte apoptosis. (**a**) Representative TUNEL staining images (magnification: 40×; scale bar: 50 μm); (**b**) Representative immunohistochemistry images of caspase-3, Bax, and Bcl-2); (**c**–**e**) Quantification of Bax, Bcl-2, and Caspase-3 protein expression; (**f**) Western blotting images of Bax, Bcl-2, and Caspase-3 proteins. (C: control group; M: CHD group; G-Rh2: ginsenoside Rh2 group; PN: PN group; P: positive control group). Compared with the M group, * *p* < 0.05, ** *p* < 0.01, *** *p* < 0.001.

TUNEL analysis (Figure 4a) revealed significantly enhanced red fluorescence densely distributed throughout the myocardial ischemic area in the M group, with some regions exhibiting patchy aggregation and nuclei appearing granular and diffuse. In contrast, the G-Rh2 group exhibited tightly arranged cells with intact nuclear membranes and minimal red fluorescence signal without any aggregated distribution. Immunohistochemical staining (Figure 4b–e) and Western blot (Figure 4f) results demonstrated that Bax and Caspase-3 protein expression levels were significantly reduced in the G-Rh2 group compared to the M Group (*p* < 0.05, *p* < 0.01), whereas the expression level of Bcl-2 was significantly elevated (*p* < 0.01). These findings suggest that G-Rh2 may attenuate pathological damage to myocardial tissue and reduce cardiomyocyte apoptosis, potentially through inhibition of Caspase-3 and Bax protein expression while promoting Bcl-2 protein expression.

### 2.4. G-Rh2 Protects Against CHD Through Lipokine Modulation and PI3K/AKT Activation

**Figure 5:**
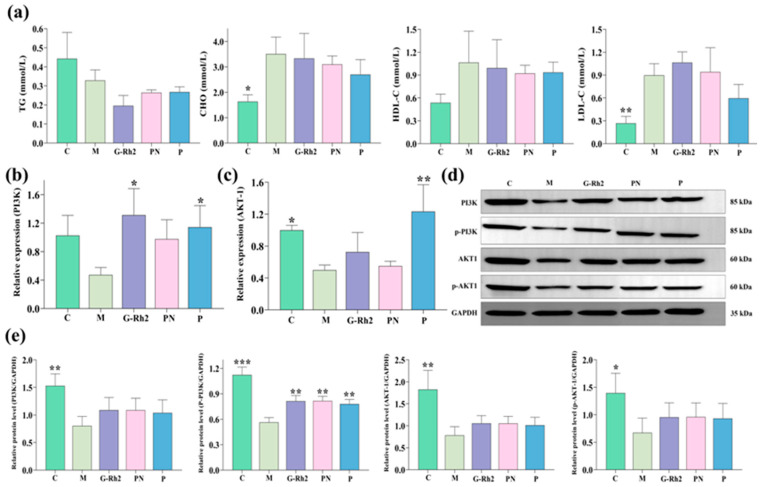
G-Rh2 regulates lipid metabolism and activates PI3K/AKT signaling in CHD rats. (**a**) Serum lipid factor levels of TG, CHO, HDL, and LDL; (**b**,**c**) mRNA expression of PI3K and AKT1; (**d**,**e**) Western blot of these proteins. (C: control group; M: CHD group; G-Rh2: ginsenoside Rh2 group; PN: PN group; P: positive control group). * *p* < 0.05, ** *p* < 0.01, *** *p* < 0.001 versus the M group.

Serum lipid analysis (Figure 5a) showed that, compared with the M group, the serum triglyceride (TG) and total cholesterol (CHO) levels in the G-Rh2 group were decreased (*p* > 0.05). Comparative analysis against normal rat serum lipid levels (CHO: 1.13–3.48 mmol/L) revealed that G-Rh2 could regulate CHO levels within the normal range, suggesting that it has a significant role in lipid homeostasis regulation and cardiovascular protective effects.

Compared to the M group, the expression of PI3K mRNA was significantly upregulated in the G-Rh2 group (*p* < 0.05, Figure 5b), with AKT1 mRNA expression exhibiting a similar trend. These results indicate that G-Rh2 upregulates PI3K and AKT1 mRNA expression in CHD rats (Figure 5c). Immunoblotting results (Figure 5d,e) showed that PI3K, p-PI3K, AKT1, and p-AKT1 protein expression was upregulated in the G-Rh2 group compared to the M group (*p* > 0.05, *p* < 0.01, *p* > 0.05, *p* > 0.05, respectively). The results indicate that G-Rh2 can upregulate the expression of PI3K and AKT1 proteins and promote their phosphorylation, thereby potentially activating the PI3K/AKT signaling cascade. This activation triggers downstream anti-apoptotic responses and myocardial cell repair, demonstrating the therapeutic potential of G-Rh2 for CHD in rats.

### 2.5. Analysis of Gut Microbiota Composition and Diversity Based on 16S rRNA Sequencing

**Figure 6:**
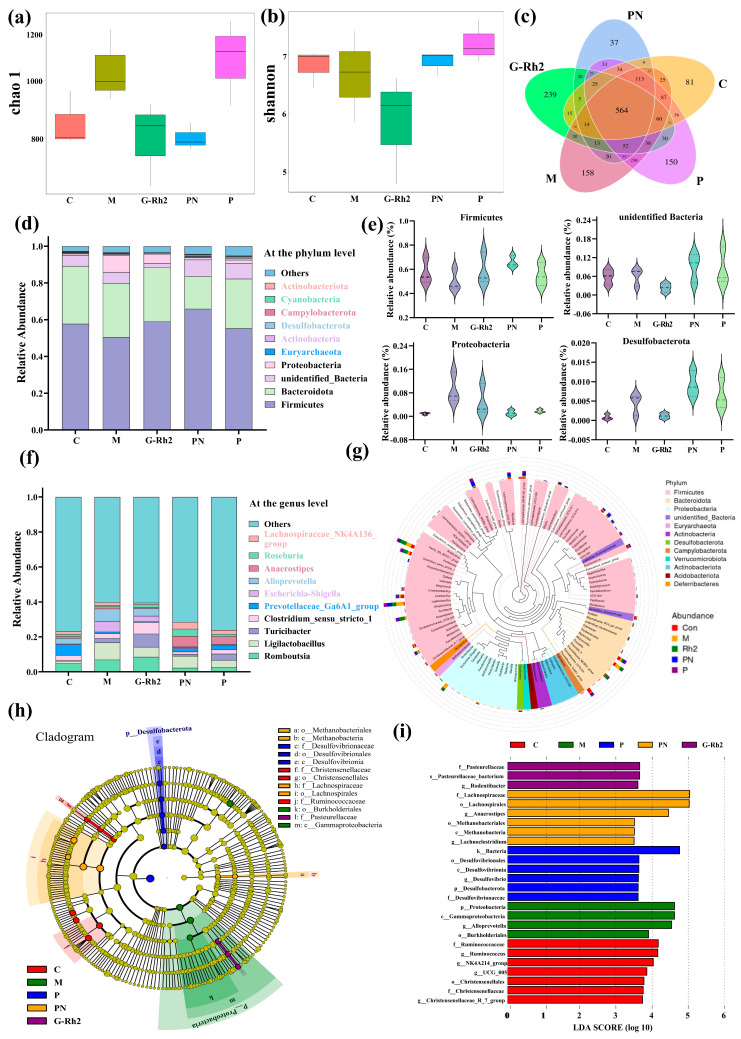
G-Rh2 reshapes gut microbiota structure and diversity in CHD rats. (**a**) Alpha diversity (Chao 1 index); (**b**) Alpha diversity (Shannon index); (**c**) Venn diagram of shared and unique OTUs; (**d**,**e**) Microbiota composition at the phylum level; (**f**) Microbiota composition at the genus level; (**g**) Dominant bacterial genera; (**h**,**i**) LEfSe analysis of differentially abundant taxa. (C: control group; M: CHD group; G-Rh2: ginsenoside Rh2 group; PN: PN group; P: positive control group).

Gut microbiota composition across all groups was analyzed using 16S rRNA amplicon sequencing, with richness and diversity assessed using alpha diversity indices (Chao1 index, Simpson index, and Shannon index). The research results indicate that CHD causes a decrease in the alpha diversity of the gut microbiota in rats (Figure 6a–c and Figure S5). Conversely, Chao1, Simpson, and Shannon indices were all elevated in the G-Rh2 group compared to the M group, indicating that G-Rh2 effectively restores gut microbiota richness and diversity caused by CHD. Taxonomic statistics were generated from clustering results, and Venn diagrams were constructed based on the OTU clustering results (Figure 6c).

At the phylum level (Figure 6d), the Firmicutes/Bacteroidetes (F/B) ratio in the M group was lower compared to the C group. At the genus level (Figure 6e,f and Figure S2), the relative abundances of species such as Romboutsia, Clostridium_sensu_stricto_1, Prevotellaceae_Ga6A1_group, and Roseburia were decreased in the M group compared to the C group, while the relative abundances of Ligilactobacillus, Turicibacter, Escherichia-Shigella, Alloprevotella, Anaerostipes, and Lachnospiraceae_NK4A136_group increased. Compared to the M group, G-Rh2 significantly decreased the relative abundances of Ligilactobacillus, Escherichia-Shigella, Alloprevotella, Anaerostipes, and Lachnospiraceae_NK4A136_group, while increasing the relative abundances of Clostridium_sensu_stricto_1 and Roseburia, indicating that G-Rh2 can regulate the gut microbiota composition in CHD rats, gradually restoring it to a normal state.

LEfSe analysis identified distinct microbial taxa across treatment groups by detecting taxonomic units with significantly different abundances (Figure 6g–i). The phylogenetic cladogram illustrated the phylogenetic distribution of these discriminative features, whereas the LDA bar chart quantified differential abundance magnitudes. The microbial consortia of the C group were primarily characterized by symbiotic bacterial taxa, including the order Christensenellales and the family Lachnospiraceae. In stark contrast, the M group exhibited significant enrichment of pathogen-associated and inflammation-related taxa, primarily within the phylum Proteobacteria, including the family Rhodocyclaceae. Following G-Rh2 intervention, unique enrichment of taxonomic units such as Veillonella and Flavobacteriaceae was observed. These taxa participate in beneficial short-chain fatty acid metabolism and anti-inflammatory processes, suggesting potential therapeutic mechanisms underlying G-Rh2 treatment.

### 2.6. Metabolomics Analysis Reveals the Regulatory Role of G-Rh2 in the Metabolic Network of CHD

**Figure 7:**
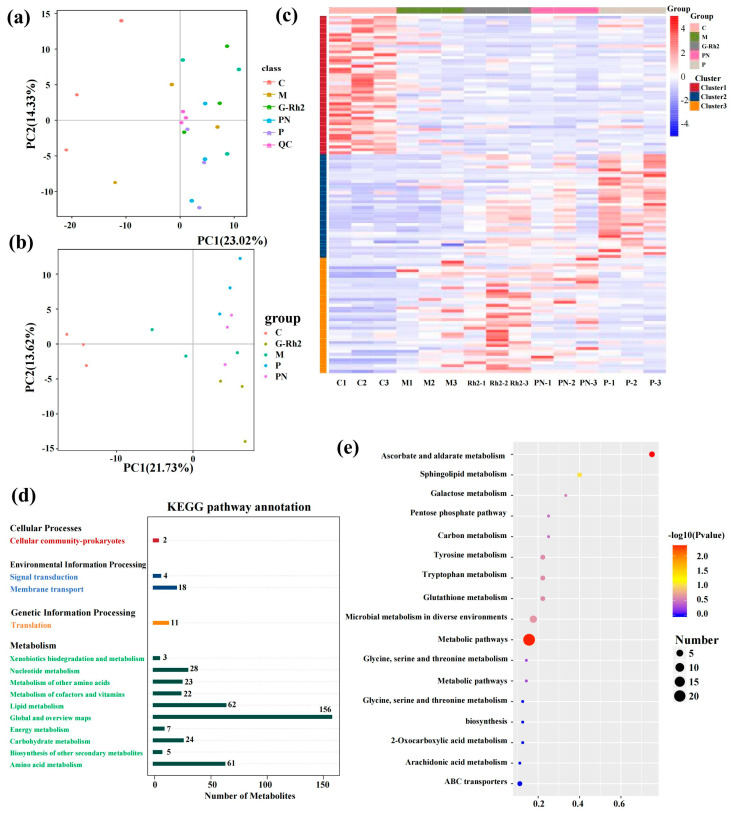
The effects of G-Rh2 on serum metabolism in rats with CHD. (**a**) PCA; (**b**) PLS-DA analysis; (**c**) Heatmap of differential metabolites among groups; (**d**) KEGG Pathway classification; (**e**) KEGG Pathway enrichment analysis.

The unsupervised PCA model showed a trend of separation between the C and M groups (Figure 7a). Subsequently, a validated PLS-DA model was established that exhibited clear separation between groups (Figure 7b). The model parameters (R^2^Y = 0.969, Q^2^ = 0.749) and the permutation test (*p* = 0.05) confirmed that the model was significant and not overfitting, demonstrating substantial metabolic differences between the groups. PCA and PLS-DA analyses (Figure S6) indicated significant differentiation between samples from the C group and the M group, with a high degree of dispersion in both groups indicative of clear differences in their metabolic profiles. QC samples exhibited distinct separation trends from the M group and other treatment groups, with QC samples clustering tightly, indicating high stability and good data quality of the LC-MS system throughout the experiment.

To further investigate metabolic differences among treatment groups, we performed PLS-DA and Student’s *t*-tests to identify differential metabolites using VIP > 1.0, |log_2_FC| > 0.263 (equivalent to FC > 1.2 or <0.833), and *p* < 0.05 as thresholds (Figure 7c). Between the C and M groups, 48 differential metabolites were identified: 27 upregulated (including lecithin, cholesterol, sphingomyelin, and acylcarnitine) and 21 downregulated (including thyroxine, L-carnitine, and isoleucine), indicating impaired lipid decomposition, disrupted energy metabolism, and enhanced atherosclerotic lesions and apoptosis. Comparison of the PN and M groups revealed 17 differential metabolites, including 12 upregulated metabolites—glycyrrhetinic acid, phosphocreatine, and valine—and 5 downregulated metabolites—glutamine and lecithin. These changes reflect the anti-inflammatory and cardioprotective effects of PN. In the P versus M comparison, 19 differential metabolites were identified: 12 were upregulated, such as phosphocreatine, phosphatidylethanolamine, and sphingosine; 7 were downregulated, including phosphatidylcholine and 13-HpOTrE(R) (hydroperoxy-octadecatrienoic acid). Finally, the G-Rh2 versus M comparison revealed 25 differential metabolites, including 15 upregulated metabolites—oleanolic acid, testosterone, phosphocreatine, and valine—and 10 downregulated metabolites, such as sphingomyelin and lecithin. These findings suggest that G-Rh2-mediated therapeutic effects on coronary heart disease may involve mechanisms related to blood glucose regulation, tissue repair, and angiogenesis promotion.

KEGG pathway enrichment analysis (Figure 7d) revealed that differential metabolites between the C group and M group were primarily enriched in pathways including amino acid biosynthesis, beta-alanine metabolism, and arginine biosynthesis. These pathways participate in the regulation of lipid metabolism and blood glucose levels. Furthermore, arginine synthesis can lower plasma concentrations of LDL and TG, contributing to atherosclerosis prevention. Differential metabolites between the M and G-Rh2 groups were mainly enriched in the pentose phosphate pathway, carbon metabolism, and pyrimidine metabolism. The intermediates of these metabolic pathways play pivotal roles in protecting against oxidative stress and DNA damage, thereby alleviating CHD-induced cardiomyocyte apoptosis.

Enrichment analysis of differential metabolites provided insights into the metabolic alterations induced by different treatments. As illustrated in Figure 7e and Figure S7, metabolic pathways affected by PN treatment relative to the M group were predominantly associated with the pentose phosphate pathway, carbon metabolism, and vitamin B6 metabolism. In contrast, the metabolic profile of the G-Rh2 group compared to the M group showed significant enrichment in the pentose phosphate pathway, carbon metabolism, and pyrimidine metabolism. The intermediate products of these pathways play pivotal roles in preventing oxidative stress and DNA damage, thereby mitigating CHD-induced cardiomyocyte apoptosis. These findings underscore that G-Rh2 may exert its cardioprotective effects by modulating key metabolic networks, reinforcing its potential as a therapeutic agent for CHD.

## 3. Discussion

CHD remains a significant clinical challenge due to the inherent limitations of current surgical and pharmacological interventions, particularly their propensity for recurrent complications, drug resistance, and adverse effects [22,23]. Against this therapeutic landscape, our investigation into G-Rh2—a rare triterpenoid saponin derived from PN—highlights its multimodal cardioprotective mechanisms that extend beyond conventional treatment paradigms [24]. Previous studies have established that G-Rh2 enhances cardiomyocyte function under conditions of oxygen-glucose deprivation, thereby preventing myocardial infarction [25]. Furthermore, G-Rh2 improves high glucose-induced cardiac dysfunction and cardiac fibrosis by modulating the PPARδ-STAT3 signaling pathway [26].

This study constructs a “drug-compound-core target-pathway” network using network pharmacology and employs molecular docking analyses to systematically investigate the role of G-Rh2 in the treatment of CHD. The findings indicate that G-Rh2 primarily exerts its therapeutic effects on CHD by modulating the activity of the PI3K/AKT signaling pathway. Molecular docking studies revealed that G-Rh2 forms stable docking conformations with core target proteins, specifically PI3K and AKT1, establishing stable hydrogen bonds and π-stacking interactions. This reveals its high bioaffinity and pharmacological activity, further supporting its potential therapeutic role in CHD. Thus, we hypothesize that G-Rh2 may exert its therapeutic effects on CHD by modulating the PI3K/AKT signaling pathway, which is implicated in the development of atherosclerosis.

Research on the molecular mechanisms of myocardial injury in coronary heart disease has demonstrated that the mitochondrial pathway of apoptosis plays a critical role in the pathological process of cardiomyocytes [27,28]. Bcl-2 family proteins (e.g., Bcl-2, Bax) mediate the release of cytochrome c (Cyt-c) by regulating mitochondrial permeability, subsequently activating the caspase cascade response [29,30]. Caspase-3 serves as the core effector molecule within this process, collectively forming a key regulatory network for cardiomyocyte apoptosis [31]. Experimental studies demonstrated that G-Rh2 modulates this apoptotic pathway through multiple targets. Specifically, it significantly downregulates the expression levels of pro-apoptotic proteins Bax and caspase-3 while upregulating the anti-apoptotic protein Bcl-2. This optimizes the Bcl-2/Bax ratio, effectively inhibiting mitochondrial Cyt-c release and blocking downstream caspase-3 activation, indicating that G-Rh2 exerts anti-apoptotic effects by regulating the mitochondrial apoptosis pathway, ultimately protecting cardiomyocytes from excessive apoptosis. In summary, these findings provide an innovative therapeutic strategy for myocardial protection in coronary heart disease.

Atherosclerosis serves as the primary pathophysiological foundation of CHD, primarily driven by disruptions in lipid metabolism [32]. Reduced levels of HDL in serum, coupled with elevated levels of CHO, LDL, and TG, are recognized as significant risk factors for atherosclerotic cardiovascular diseases [33]. Research has demonstrated that PN saponins can inhibit platelet activation through various mechanisms, thereby helping to prevent thrombosis [34]. Our findings further reveal that G-Rh2 significantly lowers serum levels of TG and CHO in rats with CHD, indicating its potential to protect myocardial tissue and enhance lipid metabolism. The PI3K/AKT pathway is crucial for regulating cardiomyocyte survival [35], angiogenesis, platelet adhesion, vascular smooth muscle cell migration, and inflammatory responses [36], making it a significant signaling pathway in the pathogenesis of various cardiovascular diseases [37]. This study demonstrates that G-Rh2 upregulates the expression of PI3K mRNA and AKT1 mRNA in rats with coronary heart disease, enhances the expression of PI3K and AKT1 proteins, and promotes their phosphorylation. These results indicate that G-Rh2 may activate the PI3K/AKT signaling pathway to exert therapeutic effects on CHD. These findings align with research by Zhao et al. on Sanqi Danshen tablets [3], Mao et al. on Aloperine [38], and Chen et al. on Salvianolic acid D [39], all of which reported therapeutic effects on CHD through modulation of the PI3K/AKT signaling pathway. However, a limitation of the current study is the lack of interventional experiments using a specific PI3K/AKT pathway inhibitor to conclusively demonstrate the necessity of this pathway for G-Rh2’s effects. Future studies employing inhibitors like LY294002 in vitro or genetic knockdown approaches are warranted to establish a definitive causal relationship. Nevertheless, our data from network pharmacology and molecular docking/dynamics collectively provide strong correlative evidence supporting the involvement of the PI3K/AKT pathway.

This study investigated the protective effects of G-Rh2 against coronary heart disease (CHD), focusing on its dual mechanisms involving modulation of the gut microbiota and metabolic pathways. Our findings indicated that G-Rh2 exerts cardiovascular protection not only through direct molecular mechanisms in the heart but also by remodeling the structure of the gut microbiota. The gut microbiota plays a crucial role in maintaining host metabolic, immune, and neural homeostasis, and its dysbiosis [40,41] is closely associated with various cardiovascular diseases, including CHD, hypertension, and heart failure [42,43]. Specifically, G-Rh2 intervention significantly reduced the relative abundance of opportunistic pathogens in the intestines of CHD rats, including Ligilactobacillus, Escherichia-Shigella, Alloprevotella, Anaerostipes, and Lachnospiraceae NK4A136_group, while promoting the increase in butyrate-producing bacteria such as Clostridium sensu stricto 1 and Roseburia. This change indicates that G-Rh2 can effectively modulate gut microbiota homeostasis, offering novel possibilities for CHD treatment. Furthermore, the microbial metabolite butyrate, identified as a key effector molecule, activates G protein-coupled receptors, promotes fatty acid oxidation in cardiomyocytes, and inhibits the NF-κB inflammatory pathway, thereby reducing cardiomyocyte apoptosis [44]. Previous studies have shown that butyrate’s effects are closely linked to the activation of the PI3K/AKT signaling pathway; it enhances the AKT phosphorylation at Ser-473 by upregulating the activity of the SIRT1 deacetylase, further promoting cardiomyocyte survival and inhibiting the expression of the fibrotic marker TGF-β1/Smad3. These findings suggest that gut microbial metabolites might participate in the functional regulation of the PI3K/AKT pathway by modulating oxidative stress [45,46,47]. These findings not only support the existence of the “gut-heart axis” but also provide new research directions for the future treatment of cardiovascular diseases.

Through metabolomics analysis, we identified 48 metabolites in the serum of normal and CHD model rats that showed significant differences, primarily involved in phospholipid metabolism, fatty acid metabolism, amino acid metabolism, and organic acid metabolism pathways. Previous studies have indicated that the downregulation of phosphatidylcholine may help alleviate coronary atherosclerosis and symptoms of blood stasis (a Traditional Chinese Medicine term referring to poor blood circulation), while fatty acid oxidation serves as a critical pathway for myocardial energy metabolism [48,49]. Amino acid metabolism is closely linked to blood stasis, particularly during the progression of CHD, where levels of metabolites such as tryptophan, arginine, and leucine are significantly elevated [50,51]. Pharmacological intervention studies demonstrated that compared to the model group, the differential metabolites in the G-Rh2 group were primarily enriched in the pentose phosphate pathway (PPP) and carbon metabolism pathways. PPP plays a vital role in regulating oxidative stress and lipid synthesis during myocardial ischemia, maintaining redox homeostasis, preventing oxidative damage, and providing nucleotide precursors necessary for myocardial repair [52,53]. G-Rh2 significantly activated PPP. Specifically, PPP reduces reactive oxygen species (ROS) levels within cardiomyocytes by supplying NADPH to maintain glutathione reductase activity. The decrease in ROS levels helps enhance the antioxidant capacity of the PI3K/AKT pathway [54,55,56]. The PPP intermediate ribose-5-phosphate activates the mTORC2 complex, promoting AKT phosphorylation at Thr-450, forming a positive feedback loop that accelerates the uptake and utilization of energy substrates (such as fatty acids and glucose) by the myocardium [44]. In summary, G-Rh2 may promote the repair of myocardial damage by regulating the pentose phosphate pathway and carbohydrate metabolism pathways, enhancing the control of oxidative stress and lipid synthesis.

## 4. Materials and Methods

### 4.1. Materials and Reagents

The (S)-G-Rh2 reference standard (Cat. NO. B21062, HPLC purity ≥ 98%) was obtained from Yunnan Yongzitang Pharmaceutical Co., Ltd. (Zhaotong, China). Although G-Rh2 is present in various Panax species, the standard used was confirmed to be sourced from PN to maintain consistency with the herbal context of this study. PN rootlet was purchased from Wenshan Miao and Zhuang Autonomous Prefecture, Yunnan Province, China (104°077′ E, 23°188′ N) and authenticated by Prof. Xiuming Cui at Kunming University of Science and Technology. The triglyceride assay kit (Lot NO. 20220822), total cholesterol assay kit (Lot NO. 20220908), high-density lipoprotein (HDL) assay kit (Lot NO. 20221025), and low-density lipoprotein (LDL) assay kit (Lot NO. 20221010) were all procured from Nanjing Jiancheng Bioengineering Institute Co., Ltd. (Nanjing, China). The PI3K antibody (Cat. NO. AF6241), phospho-PI3K antibody (Cat. NO. AF3242), AKT antibody (Cat. NO. AF0836), and phospho-AKT antibody (Cat. NO. AF0832) were obtained from Affinity Biosciences. The real-time quantitative polymerase chain reaction (RT-qPCR) kit was obtained from Tiangen Biochemical Technology Co., Ltd. (Beijing, China) (Cat. NO. RK145). The β-actin antibody (Cat. NO. AB2839417), anti-rabbit secondary antibody (Cat. NO. AB2839429), and anti-mouse secondary antibody (Cat. NO. AB2839430) were sourced from Affinity Bioscience (Shanghai, China).

### 4.2. Network Pharmacology Analysis and Molecular Docking

#### 4.2.1. Network Pharmacology Analysis

Prediction targets of G-Rh2 were performed using three major targeting prediction platforms: SuperPred (https://prediction.charite.de/ accessed on 6 May 2026, species: Human), SEA v1.5 (https://sea.bkslab.org/, species: Human, *p*-value < 1 × 10^−10^ and MaxTC > 0.5), and Swiss Target Prediction v1.0 (http://www.swisstargetprediction.ch/, accessed on 25 April 2023, Probability > 0.1). These results were integrated with data retrieved from the HERB database (http://47.92.70.12), OMIM (https://omim.org/), and GeneCards (https://www.genecards.org/) to construct a comprehensive gene set associated with CHD [21]. A Venn diagram analysis was subsequently performed using the Venn Diagram 1.10 tool to identify the overlapping targets of G-Rh2 relevant to CHD treatment.

The STRING database (version 11.5) was employed to construct a protein–protein interaction (PPI) network for the candidate target proteins, with the organism set to Homo sapiens and the minimum required interaction score set to 0.400, which resulted in the generation of a PPI network file. Cytoscape 3.9.0 was then used to visualize the herb-target-pathway ternary interaction network, and core targets were identified based on their degree values within the PPI network [57].

R (version 4.2.2) and an online bioinformatics visualization platform were employed for Gene Ontology (GO) and Kyoto Encyclopedia of Genes and Genomes (KEGG) enrichment analysis of the overlapping targets between traditional Chinese medicine and disease-associated genes. Bar charts were generated to display GO and KEGG classification results based on the number of enriched genes, and the core signaling pathways were visualized accordingly [58].

#### 4.2.2. Molecular Docking Validation

Based on the therapeutic targets and pathways for CHD identified through network pharmacology analysis, molecular docking was performed using computational pharmacology methods. The three-dimensional (3D) structure file of G-Rh2 (PubChem CID:119307) was obtained from the PubMed database (https://pubmed.ncbi.nlm.nih.gov/, accessed on 12 May 2023), and structure optimization was conducted using OpenBabel (version 3.1.1). The MMFF94 force field was applied for energy minimization to obtain the lowest-energy 3D coordinate parameters. Molecular docking was subsequently performed using AutoDock Vina (version 1.2.0) to calculate binding free energies, and the resulting docking conformations were visualized and analyzed using PyMOL (version 2.3.0).

#### 4.2.3. Molecular Dynamics Simulation

Molecular dynamics simulations were performed using GROMACS (version 2024.4) to analyze the AKT1 protein (PDB ID: 3cqu), PI3K protein (PDB ID: 4JPS), and their respective complexes with the ligand ginsenoside Rh2. The Amber14SB force field was applied to proteins, and the GAFF2 force field was used for the ligand. The TIP4P water model was used for solvation, with periodic boundary conditions applied in a cubic water box extending 1.2 nm beyond the solute surface. Long-range electrostatic interactions were calculated using the Particle Mesh Ewald (PME) method. Sodium and chloride ions were introduced via the Monte Carlo method to neutralize the system charge, followed by energy minimization and equilibration. Subsequently, 100 ns unrestrained production runs were conducted using a 2 fs timestep, with coordinates saved every 10 ps. Trajectory analysis included root mean square deviation (RMSD), root mean square fluctuation (RMSF), radius of gyration (Rg), hydrogen bond counts, free energy landscapes, and structural comparisons of snapshots at 0, 25, 50, 75, and 100 ns. Protein-ligand binding free energies were calculated using the MM/GBSA methodology.

### 4.3. Animal Experiments

#### 4.3.1. Experimental Animals

Seventy SPF-grade male Wistar rats (6 weeks old, 180–200 g) were purchased from Beijing Spifei Biotechnology Co., Ltd. (Beijing, China) (License No. SCXK [Jing] 2019-0010) and housed at the Animal Experiment Center of Kunming University of Science and Technology for a 7-day acclimatization period (housing conditions: temperature 25 ± 1 °C; humidity 45–55%; 12 h dark/light cycle). All experimental protocols complied with the guidelines of the National Institutes of Health and were approved by the Animal Ethics Committee of Kunming University of Science and Technology (license No. SYXK [Dian] K2022-0001).

#### 4.3.2. Establishing the CHD Model and Sample Collection

The 70 rats were randomly divided into two groups: the control group (group C, *n* = 8) and the CHD model group (*n* = 62). The CHD model group was initially fed a high-fat diet (30 g/day) containing 10% yolk powder, 2% cholesterol, 10% lard oil, 0.2% propylthiouracil, 0.5% sodium cholate, and 77.3% standard chow for 10 weeks. Pituitrin (30 U/kg, National Drug Approval Number: 32026637, Batch Number: 25251101) was intraperitoneally injected twice daily for the two days preceding the final feeding [59]. After 60 days, 32 rats had successfully developed CHD (Figure 1, Figure 3a and Figure S1). These model rats were then randomly divided into four groups (*n* = 8/group): the model group (group M, 0.9% saline, 5 mL/kg/day), the G-Rh2 group (10 mg/kg/day), the PN group (200 mg/kg), and the positive control group (group P; compound Danshen dripping pill aqueous solution, 80 mg/kg/day), all administered via gavage. Concurrently, the control group received 0.9% saline (5 mL/kg) via gavage for 4 weeks.

After the final administration, the rats were anesthetized with ether, and arterial blood was collected. The blood was centrifuged (4 °C, 12,000 rpm, 15 min) to obtain the supernatant, which was stored at −80 °C for subsequent analysis. Following euthanasia by cervical dislocation, the heart was excised and washed twice with 0.9% saline.

#### 4.3.3. Hematoxylin-Eosin (H&E) and Masson Staining

Heart samples were fixed in 4% paraformaldehyde for 24 h, embedded in paraffin, and sectioned at 3–4 μm using a microtome. The sections were subjected to standard H&E staining procedures, including deparaffinization, hydration, hematoxylin staining, eosin staining, dehydration, and mounting. The stained sections were scanned using a digital slide scanner and analyzed with SlideViewer 2.5 software.

For Masson’s trichrome staining, paraffin-embedded sections were deparaffinized, rehydrated, and stained with Weigert’s iron hematoxylin, followed by differentiation in acidic ethanol, bluing in Masson’s blue solution, and counterstaining with Biebrich scarlet and aniline blue solutions. After dehydration, the slides were mounted and scanned using the same digital slide scanner and analyzed with SlideViewer software.

#### 4.3.4. Detection of Serum Lipid Factor Levels

According to the methods described in the manuals for the triglyceride (TG) assay kit, cholesterol (CHO) assay kit, high-density lipoprotein (HDL) assay kit, and low-density lipoprotein (LDL) assay kit, the content of serum lipid factors in rats was measured using a fully automated biochemical analyzer (Chemray 800, Guangdong, China).

#### 4.3.5. TUNEL Staining and Immunohistochemical Analysis

Apoptosis in cardiomyocytes was detected using a TUNEL assay kit (Cat. NO. KTA2011, Wuhan, China). Tissue sections were deparaffinized, rehydrated, and digested with proteinase K, followed by incubation with the TUNEL reaction mixture. After counterstaining with DAPI, the sections were mounted with anti-fade medium and examined under a fluorescence microscope.

Paraffin-embedded tissue sections were deparaffinized, rehydrated, and subjected to antigen retrieval using citrate buffer (pH 6.0). Endogenous peroxidase activity was quenched with 3% hydrogen peroxide, and nonspecific binding sites were blocked with 3% bovine serum albumin (BSA). The sections were then incubated overnight at 4 °C with primary antibodies against caspase-3, Bax, and Bcl-2, followed by incubation with horseradish peroxidase (HRP)-conjugated secondary antibodies. After counterstaining, dehydration, and clearing, the sections were mounted and examined under a laser scanning confocal microscope.

### 4.4. RT-qPCR and Western Blotting Analysis

Total RNA was extracted using TRNzol Universal Reagent and reverse transcribed into cDNA using the StarScript kit (Cat. NO. A240-02, Beijing, China). Quantitative real-time PCR (RT-qPCR) was performed using gene-specific primers (Table S1), with GAPDH serving as the internal reference. Relative gene expression was calculated using the 2^−ΔΔCt^ method.

Total protein was extracted using RIPA lysis buffer, and protein concentrations were determined using a BCA Protein Quantification Kit. Equal amounts of protein were separated by SDS-PAGE and transferred to PVDF membranes for immunoblotting. Protein bands were visualized using enhanced chemiluminescence (ECL) and quantified by densitometric analysis using ImageJ software(Version 1.54p).

### 4.5. Gut Microbiome Analysis

Cecal contents were collected in sterile centrifuge tubes and stored at −80 °C. DNA was extracted using the QIAamp DNA Stool Mini Kit (Cat. NO. 51604, Qiagen, North Rhine-Westphalia, Germany) according to the manufacturer’s instructions. DNA quality was assessed by agarose gel electrophoresis, and DNA concentration and purity were measured using a microvolume UV spectrophotometer (EzDrop 1000, Hangzhou, China). The V3–V4 region of the 16S rRNA gene was amplified by PCR and sequenced on the NovaSeq 6000 platform (Illumina, Shanghai, China).

### 4.6. Metabolomics Analysis

Serum metabolites were analyzed using ultra-high performance liquid chromatography tandem mass spectrometry (UPLC-MS/MS). Quality control (QC) was included to ensure data reliability. Data were acquired using Xcalibur 4.1 software (Thermo Fisher Scientific, Waltham, MA, USA). Principal component analysis (PCA) was performed to assess overall metabolic variation among samples by SIMCA software (Version 16.0.2). Partial least squares discriminant analysis (PLS-DA) and orthogonal PLS-DA (OPLS-DA) were conducted to identify differential metabolites. Model validity was assessed by permutation testing (*n* = 200), with *p* < 0.05 indicating statistical significance. Differential metabolites were selected based on variable importance in projection (VIP) scores > 1 from the OPLS-DA model and *p* < 0.05 from Student’s *t*-test. Metabolite annotation and pathway enrichment analysis were performed using the Kyoto Encyclopedia of Genes and Genomes (KEGG) database. Pathway enrichment was analyzed using the SciPy 1.12 Python package, with significance determined by Fisher’s exact test.

### 4.7. Statistical Analysis

All experiments were carried out in triplicate, and all data are presented as mean ± standard deviation (SD). Statistical significance was determined by two-way ANOVA followed by Tukey’s Honestly Significant Difference (HSD) test, with *p* < 0.05 considered statistically significant.

## 5. Conclusions

In this study, comprehensive analysis using network pharmacology and molecular docking techniques revealed that the PI3K/AKT signaling pathway may be the main signaling pathway through which G-Rh2 exerts its therapeutic effects against CHD. Molecular docking further validated the stable binding of G-Rh2 with core targets (PI3K and AKT1), elucidating the underlying molecular mechanisms. Additionally, pharmacological and metabolomics results indicate that G-Rh2 ameliorates CHD through multidimensional approaches, including the regulation of lipid metabolism, regulation of PI3K/AKT protein phosphorylation, modulation of metabolic pathways, and improvement of gut microbiota balance. These findings provide novel insights and potential therapeutic strategies for the prevention and treatment of CHD.

## Data Availability

The data presented in this study are available on request from the corresponding authors due to institutional restrictions and ongoing research.

## Supplementary Materials

The supporting information can be downloaded at https://www.mdpi.com/article/10.3390/ijms27115133/s1.

## Abbreviations

The following abbreviations are used in this manuscript:

AKT	Protein Kinase B
BCA	Bicinchoninic Acid Assay
BP	Biological Processes
BSA	Bovine Serum Albumin
CABG	Coronary Artery Bypass Grafting
CC	Cellular Components
cDNA	Complementary DNA
CHD	Coronary Heart Disease
CHO	Cholesterol
CK-MB	Creatine Kinase-MB
cTnI	Cardiac Troponin I
Cyt-c	Cytochrome c
ECG	Electrocardiogram
EF	Ejection Fraction
F/B	Firmicutes/Bacteroidetes
FC	Fold Change
FS	Fractional Shortening
GAPDH	Glyceraldehyde-3-Phosphate Dehydrogenase
G-Rh2	Ginsenoside Rh2
H&E	Hematoxylin-Eosin
HDL	High-Density Lipoprotein
HFD	High-Fat Diet
HRP	Horseradish Peroxidase
KEGG	Kyoto Encyclopedia of Genes and Genomes
LDL	Low-Density Lipoprotein
MF	Molecular Functions
NADPH	Nicotinamide Adenine Dinucleotide Phosphate
OPLS-DA	Orthogonal Partial Least Squares Discriminant Analysis
PCA	Principal Component Analysis
PCI	Percutaneous Coronary Intervention
PI3K	Phosphatidylinositol-3-Kinase
PLS-DA	Partial Least Squares Discriminant Analysis
PN	*Panax* *notoginseng*
PPI	Protein–Protein Interaction
PPP	Pentose Phosphate Pathway
QC	Quality Control
RIPA	Radio Immunoprecipitation Assay Lysis Buffer
ROS	Reactive Oxygen Species
RT-qPCR	Quantitative Reverse Transcription Polymerase Chain Reaction
TG	Triglyceride
