# Supplementary material for: Ginsenoside Rh2 Regulates PI3K/AKT Signaling, Metabolic Pathways, and the Gut Microbiota for Coronary Heart Disease Therapy
Source: Int J Mol Sci. 2026 Jun 5;27(11):5133. doi: 10.3390/ijms27115133 (PMC13257083; doi:10.3390/ijms27115133)
Supplement: Supplementary file 1 [file ijms-27-05133-s001.zip › ijms-4333386-supplementary.pdf]

Electrocardiogram (ECG) Detection in CHD Rats: The rats were anesthetized using an intraperitoneal injection of 1% pentobarbital sodium (0.3 mL/100g) and subsequently fixed onto the experimental platform. Following successful anesthesia, electrodes were carefully inserted subcutaneously into the limbs and chest of each rat according to the designated lead placement scheme: the right upper limb was designated red (R), the left upper limb yellow (L), the left lower limb green (F), the right lower limb black (RF), and the chest lead was indicated by a red-and-white pattern (C1). The lead wires were then connected to a digital multi-channel veterinary ECG device (ZoncarE, Imac 300VET), facilitating the recording of the electrocardiogram.

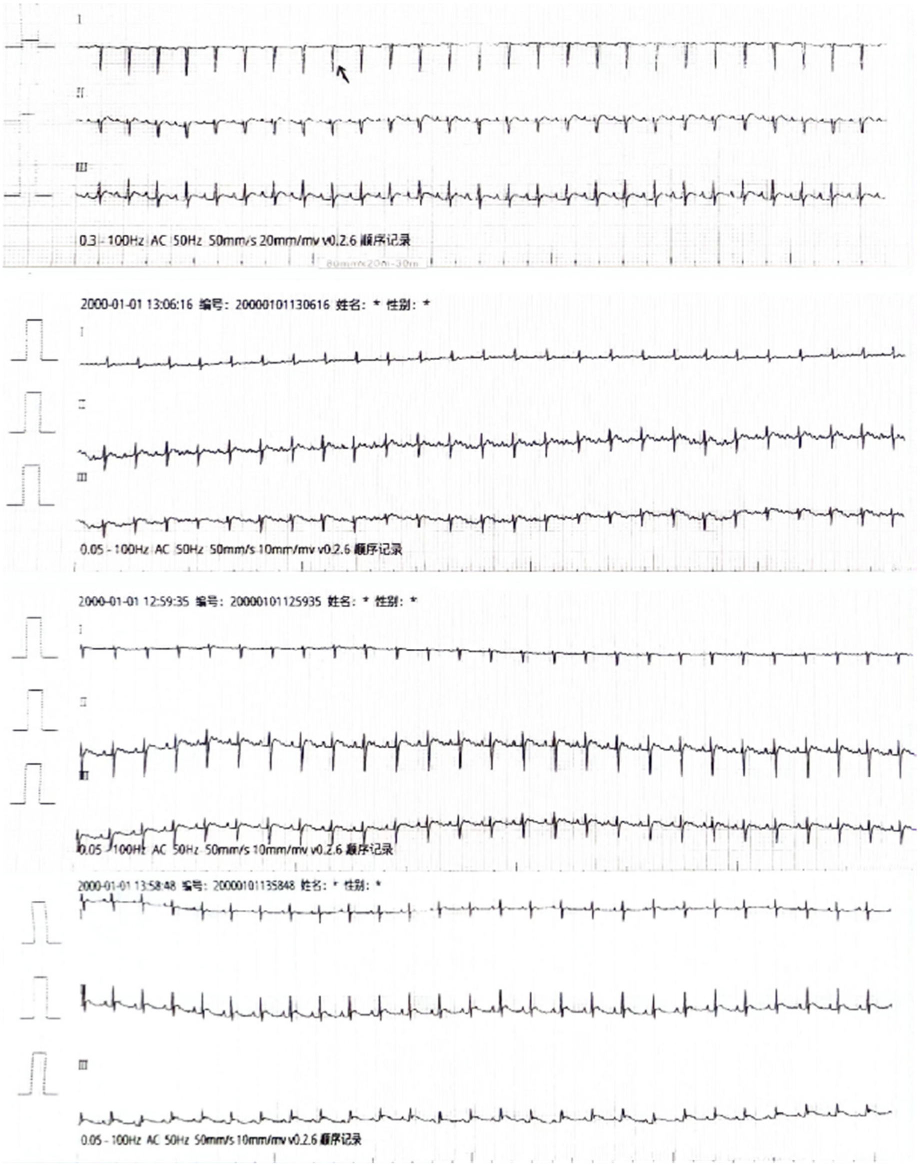

Figure S1. Coronary heart disease model of electrocardiogram (ECG).

Table S1 primer sequences list

Gene	primer sequences Forward/Reverse（5’-3’）
PI3K	F: TGGCTTACGCTCCAGTATTTGC<br>R: TAGAAGTGGGCTTGGGTGGTTTA
AKT1	F: AGTCCCCACTCAACAACTTCT<br>R: GAAGGTGCGCTCAATGACTG
GAPDH	F: CTGGAGAAACCTGCCAAGTATG<br>R: GGTGGAAGAATGGGAGTTGCT

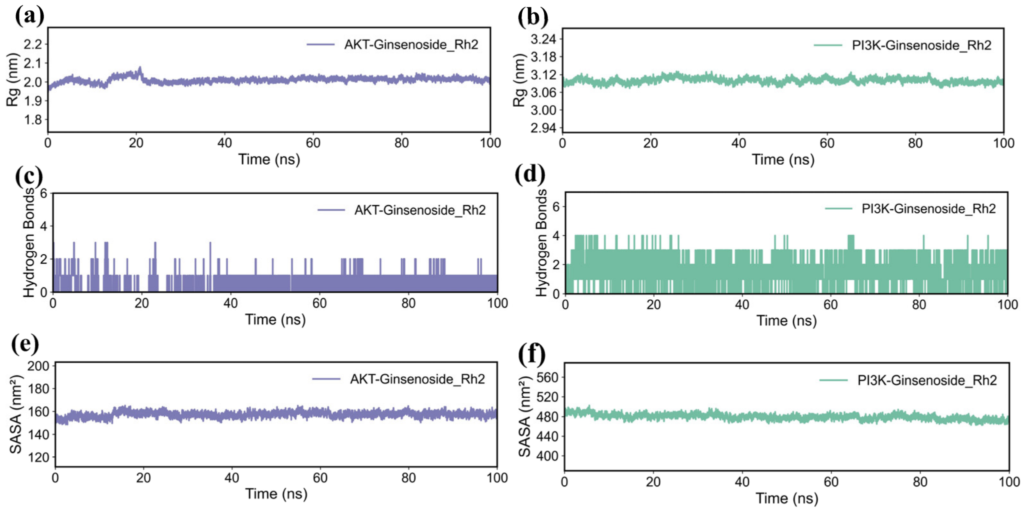

Figure S2. For Radius of Gyration (Rg) Profiles: (a) AKT1 complex, (b) PI3K complex. For Hydrogen Bond Fluctuation Profiles: (c) AKT1-ligand interactions, (d) PI3K-ligand interactions. For Solvent Accessible Surface Area (SASA) Profiles: (e) AKT1 complex, (f) PI3K complex.

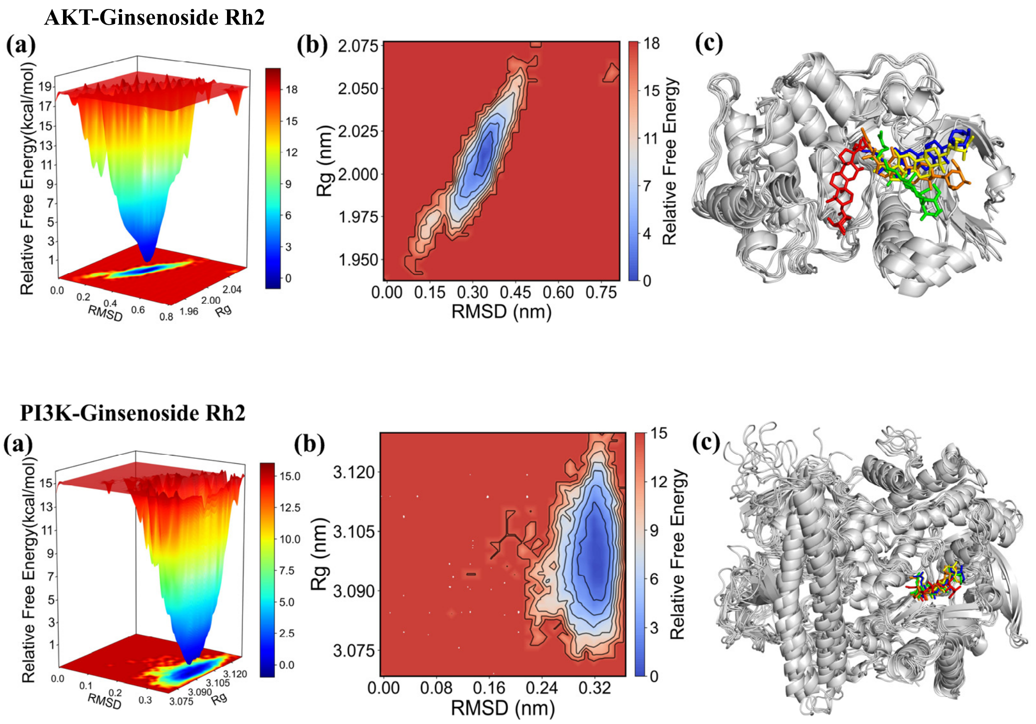

Figure S3. For AKT1-Ginsenoside-Rh2 Complex: (a-b) Free energy landscape; (c) Structural comparison at 0, 25, 50, 75, and 100 ns. Red, green, blue, yellow, and orange depict Ginsenoside-Rh2 conformations at 0, 25, 50, 75, and 100 ns, respectively. For PI3K-Ginsenoside-Rh2 Complex: (a-b) Free energy landscape; (c) Structural comparison at 0, 25, 50, 75, and 100 ns. Red, green, blue, yellow, and orange represent Ginsenoside-Rh2 conformations at 0, 25, 50, 75, and 100 ns, respectively.

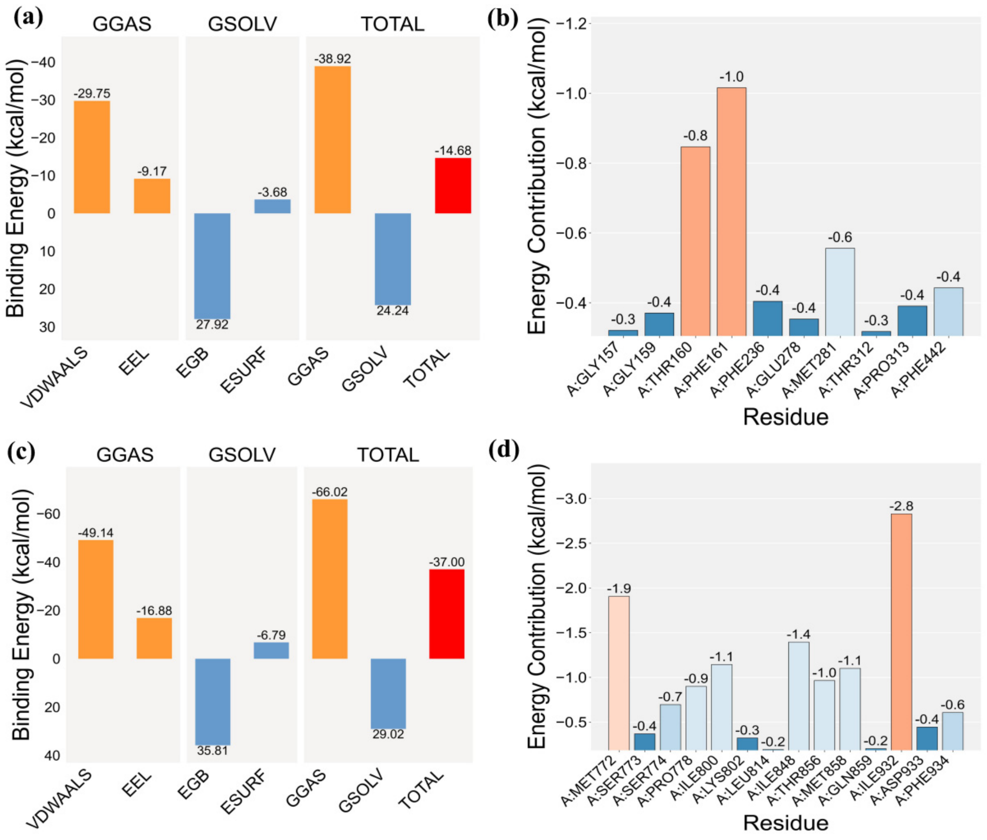

Figure S4. Average binding free energy: (a) AKT1 complex, (b) PI3K complex. VDWAALS: Van der Waals forces; EEL: Coulombic electrostatic energy; EGB: Polar solvation energy; ESURF: Nonpolar solvation energy; GGAS: Gas-phase free energy; GSOLV: Solvation free energy; TOTAL: Total binding free energy. Residue energy decomposition profiles: (c) AKT1 complex, (d) PI3K complex. Represent energy contributions of amino acid residues participating in Ginsenoside-Rh2 binding.

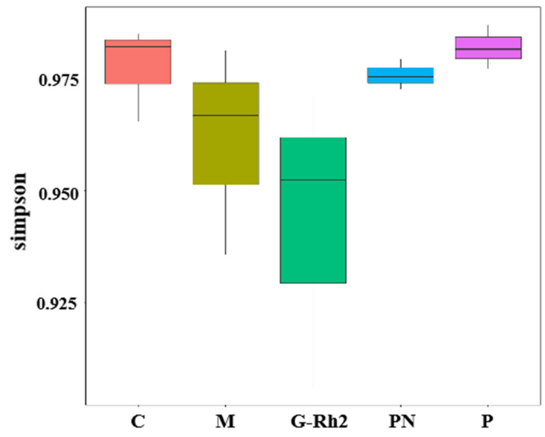

Figure S5. Effects of G-Rh2 on the gut microbiota structure in fecal samples.

α-diversity-simpson.

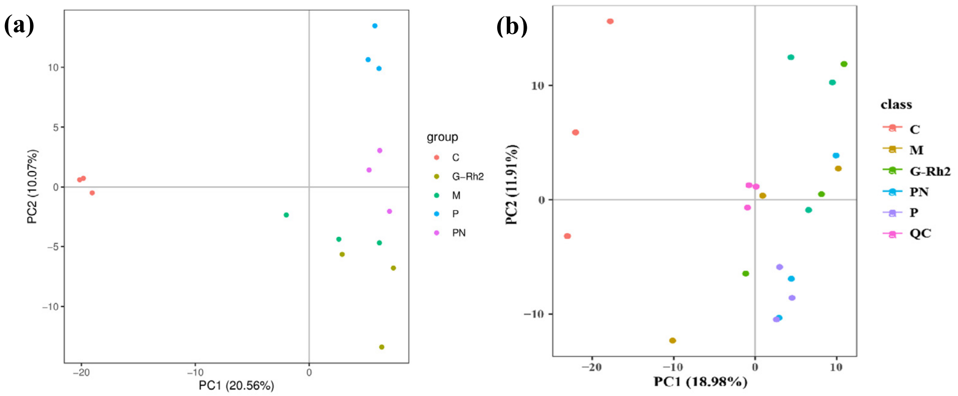

Figure S6. The effects of G-Rh2 on serum metabolism in rats with CHD. (a) PCA analysis; (b) PLS-DA analysis

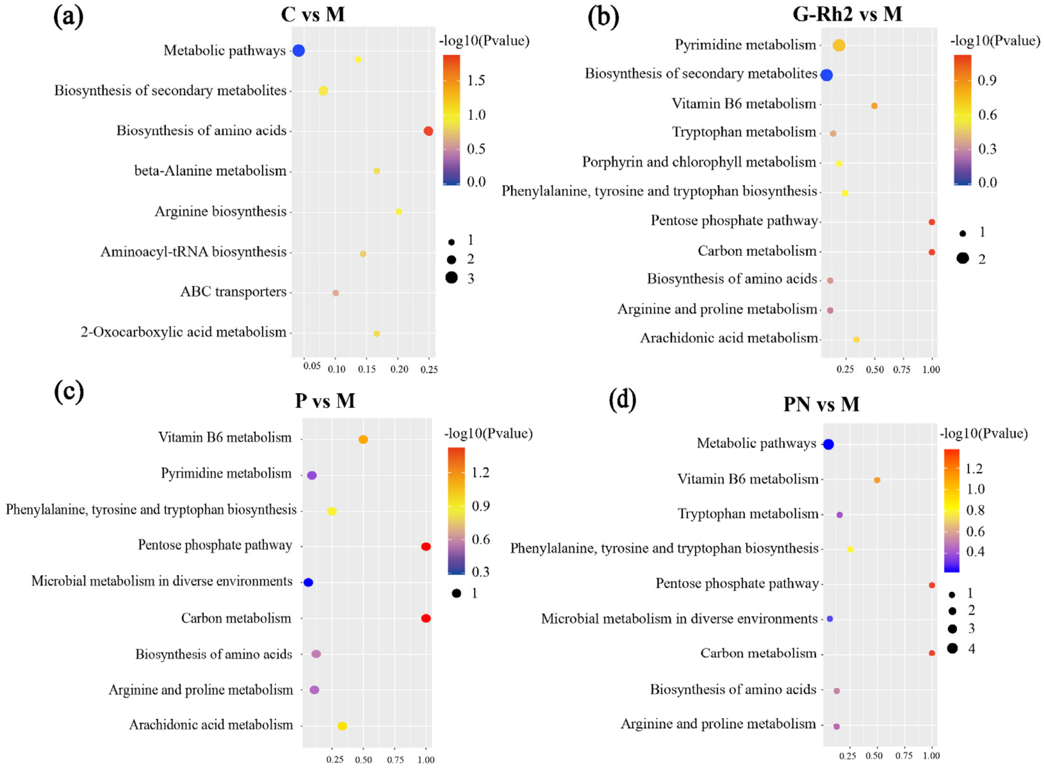

Figure S7. Differential metabolite enrichment pathways. (a) Group C vs Group M; (b) Group M vs Group G-Rh2; (c) Group M vs Group P; (d) Group M vs Group PN. (C: control group; M: CHD group; G-Rh2: Ginsenoside-Rh2 group; PN: PN group; P: positive control group).
